# A model for the compositions of non-stoichiometric intermediate phases formed by diffusion reactions, and its application to Nb_3_Sn superconductors

**DOI:** 10.1038/srep19096

**Published:** 2016-01-12

**Authors:** X. Xu, M. D. Sumption

**Affiliations:** 1 Department of Materials Science and Engineering, the Ohio State University, Columbus, OH 43210 USA

## Abstract

In this work we explore the compositions of non-stoichiometric intermediate phases formed by diffusion reactions: a mathematical framework is developed and tested against the specific case of Nb_3_Sn superconductors. In the first part, the governing equations for the bulk diffusion and inter-phase interface reactions during the growth of a compound are derived, numerical solutions to which give both the composition profile and growth rate of the compound layer. The analytic solutions are obtained with certain approximations made. In the second part, we explain an effect that the composition characteristics of compounds can be quite different depending on whether it is the bulk diffusion or grain boundary diffusion that dominates in the compounds, and that “frozen” bulk diffusion leads to unique composition characteristics that the bulk composition of a compound layer remains unchanged after its initial formation instead of varying with the diffusion reaction system; here the model is modified for the case of grain boundary diffusion. Finally, we apply this model to the Nb_3_Sn superconductors and propose approaches to control their compositions.

## Introduction

Intermediate phases with finite composition ranges represent a large class of materials, and their compositions may influence their performance in application, as demonstrated in a variety of materials, such as electrical conductivity of oxides (e.g., TiO_2−y_^1^), electromagnetic properties of superconductors (e.g., Nb_3_Sn and YBa_2_Cu_3_O_7−y_^2^), and mechanical properties of some intermetallics (e.g., Ni–Al_0.4–0.55_^3^), etc. For instance, the superconducting Nb_3_Sn phase, which finds significant applications in the construction of 12–20 T magnets^4^,^5^,^6^, has a composition range of ~17–26 Sn at.%^7^,^8^, and its superconducting transition temperature and magnetic field decrease dramatically as Sn content drops^4^,^8^,^9^. The Nb_3_Sn phase, which is generally fabricated from Cu-Sn and Nb precursors through reactive diffusion processes, is always found to be Sn-poor (e.g., 21–24 at.%^4^,^9^,^10^), making composition control one of the primary concerns in Nb_3_Sn development since 1970s^10^. Although a large number of previous experiments (e.g.,^4^,^9^,^10^,^11^,^12^) have uncovered some factors that influence the Sn content, it is still a puzzle what fundamentally determines the Nb_3_Sn composition. This work aims to fill that gap. Here it is worth mentioning that the composition interval of a compound layer does not necessarily coincide with its equilibrium phase field ranges – the former can be narrower (e.g., the Nb_3_Sn example above) if the inter-phase interface reaction rates are slow relative to the diffusion rate across the compound, which results in discontinuities in chemical potentials at the interfaces.

There have been numerous studies regarding diffusion reaction processes, most of which have focused on layer growth kinetics (e.g.,^13^,^14^,^15^,^16^,^17^), compound formation and instability (e.g.,^15^,^16^,^17^), phase diagram determination (e.g.,^18^), and interdiffusion coefficient measurement (e.g.,^19^), while a systematic model exploring how to control compound composition is still lacking. We find it indeed possible to modify the model developed by Gosele and Tu^14^ for deriving the layer growth kinetics of compounds to calculate their compositions; however, certain assumptions (e.g., steady-state diffusion and first-order interface reaction rates) that the model was based on may limit the accuracy of the composition results. In this work, we aim to develop a rigorous, systematic mathematical framework for the compositions of intermediate phases.

### The model

**Figure 1:**
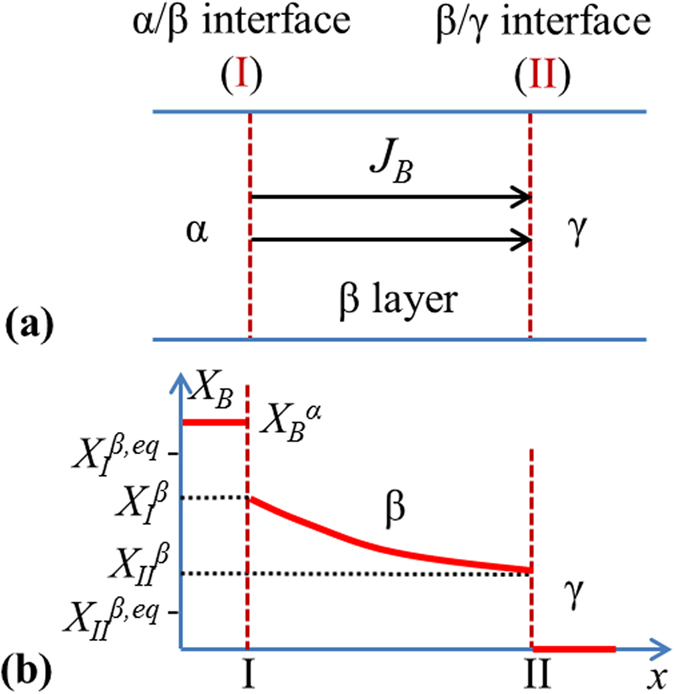
Schematics of (a) the α/β/γ diffusion reaction system in the planar geometry, and (b) *X*_*B*_ profiles of the system.

**Figure 2:**
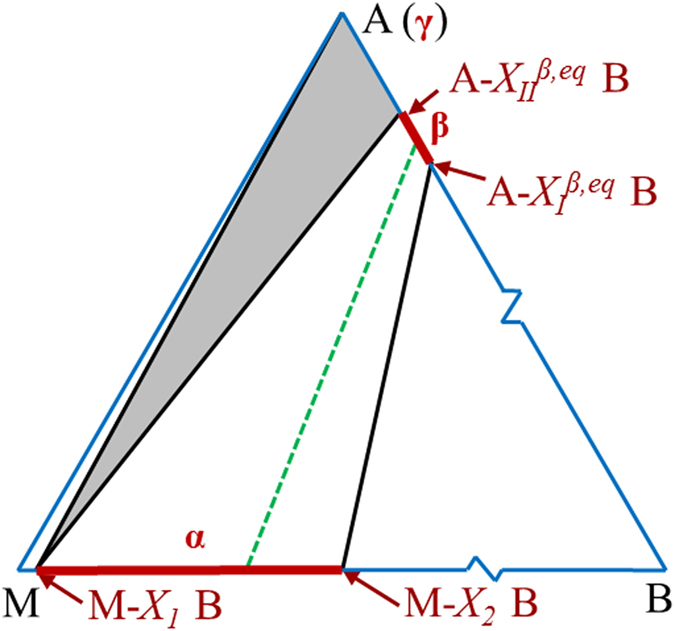
Schematic of an isothermal cross section of the M–A–B ternary phase diagram. The shaded region shows the equilibria among M-*X*_*1*_ B, A-*X*_*I*_^*β,eq*^ B, and γ phases, and the dashed line shows the equilibrium between α and β phases.

Let us consider that a non-stoichiometric A_n_B compound is formed in a system of M–B/A, where M is a third element that does not dissolve in A_n_B lattice^20^. The use of the third element M is to decrease the chemical potential of B, so that unwanted high-B A–B compounds (e.g., NbSn_2_ and Nb_6_Sn_5_ in the Nb-Sn system^7^) that would form in the B/A binary system can be avoided. With the M–B, A_n_B, and A-rich phases denoted as α, β, and γ, respectively, a schematic of the α/β/γ system for a planar geometry is shown in Fig. 1. Let us denote the α/β and β/γ inter-phase interfaces as I and II, respectively, and the mole fractions, chemical potentials, activities, and diffusion fluxes of B in the β phase at interfaces I and II as *X*_*I*_^*β*^, *μ*_*I*_^*β*^, *a*_*I*_^*β*^, *J*_*I*_^*β*^, and *X*_*II*_^*β*^, *μ*_*II*_^*β*^, *a*_*II*_^*β*^, *J*_*II*_^*β*^, respectively. The maximum and minimum mole fractions of B in β phase (i.e., A_n_B compound) from the phase diagram are set as *X*_*I*_^*β,eq*^ and *X*_*II*_^*β,eq*^, respectively. Let us also denote the *μ*_*B*_s and *a*_*B*_s of α and γ as *μ*_*B*_^α^, *a*_*B*_^α^, and *μ*_*B*_^γ^, *a*_*B*_^γ^, respectively. Let us assume that the solubility of B in γ phase is negligible^7^. An isothermal cross section of such an M–A–B phase diagram at a certain temperature is shown in Fig. 2. This is the case we see for the Nb_3_Sn example above (for which A stands for Nb, B for Sn, and M for Cu), but the model below can be modified for other cases. Similar to the Cu-Nb-Sn system, let us assume B is the primary diffusing species in the β phase^21^ and that the α phase can act as an intensive sink for B vacancies in order for it to be an efficient source of B atoms for β layer growth, and that the diffusivity of B in α is high so that the α phase remains homogeneous during the growth of β layer^11^.

Here we assume that diffusion occurs by vacancy mechanism and the total atomic flux is balanced by the vacancy flux. As discussed in the papers by Svoboda and Fischer *et al.*^22^,^23^,^24^, the presence of various types of sinks or sources for vacancies may lead to quite different diffusional and conservation laws and equations. For this model, we assume that B vacancies are generated by the reaction at interface II (as will be discussed in detail later), and then diffuse across the β layer to interface I, where they are annihilated by B atoms from α phase (the B source). For the simplicity of the model, we assume that there are no sinks or sources for vacancies in the bulk or grain boundaries of β phase, while the only sink in the system for B vacancies is the α phase. The following model can be modified for cases with other types of sinks or sources for vacancies using the models by Svoboda and Fischer *et al.*^22^,^23^,^24^.

In this work let us assume the diffusivity of B in β phase, *D*, and the molar volume of β phase, *V*_*m*_^β^, do not vary with *X*_*B*_, in which case the continuity equation in the A_n_B layer is given by:


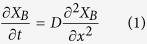


According to mass conservation, in a unit time the amount of B transferring across the interface I should equal to that diffusing into the β layer from the interface I, and the amount arriving at the interface II should equal to that transferring across it, i.e., *dn*/*dt*|_I_  = *J*_*I*_^β^∙*A*_*I*_, and *dn*/*dt*|_II_ = *J*_*II*_^β^∙*A*_*II*_, where *A*_*I*_ and *A*_*II*_ are the areas of the interfaces I and II, respectively. The molar transport rate *dn*/*dt* across an interface equals to *r*∙*A*_*int*_∙exp(−*Q*/*RT*)∙[1−exp(−Δ*μ*/*RT*)], where *r* is the transfer rate constant for this interface with the unit of mol/(m^2^∙s), *A*_*int*_ is the interface area, *Q* is the energy barrier, *R* is the gas constant, *T* is the temperature in K, and Δ*μ* is the driving force for atom transfer. For the interface I, Δ*μ*|_I_ = *μ*_*B*_^*α*^−*μ*_*I*_^*β*^. For the interface II, Δ*μ*|_II_ = *μ*_*II*_^*β*^–*μ*_*B*_^*γ*^, and *μ*_*B*_^*γ*^ = *μ*_*B*_(A−*X*_*II*_^*β,eq*^ B). With *J*_*B*_ = −(*D*/*V*_*m*_)∙(∂*X*_B_/∂*x*), we have:


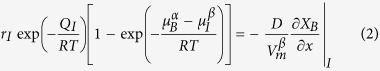



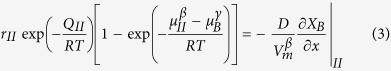


Eqs. (2) and (3) are the boundary conditions for Eq. (1). Note that *X*_*B*_ in α phase, *X*_*B*_^*α*^, drops with annealing time as B in α is used for β layer growth, so *μ*_*B*_^*α*^ drops with *t*:


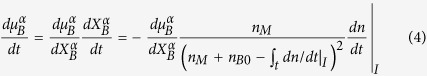


where *n*_*M*_ and *n*_*B0*_ are the moles of M and B in the M-B precursor. For those systems without the third element, *μ*_*B*_^*α*^ is constant, and Eq. (4) is not needed. In addition, since the B atoms diffusing to the interface II are used to form new β layers, we have:


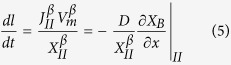


Eqs. (1)–(5) are the governing equations for the system set up above, solutions to which give both the *X*_*B*_(*x*, *t*) and the *l*(*t*) of a growing A_n_B layer. It should be noted that for the systems with large volume expansion associated with transformation from γ to β, stress effects need to be considered^25^.

To simplify Eqs. (2) and (3), we notice that 1−exp[−(*μ*_*B*_^*α*^−*μ*_*I*_^*β*^)/*RT*] = 1−*a*_*I*_^*β*^/*a*_*B*_^*α*^, since *μ*_*B*_^*α*^−*μ*_*I*_^*β*^ = *RT*ln(*a*_*B*_^*α*^/*a*_*I*_^*β*^); similarly, 1−exp[−(*μ*_*II*_^*β*^−*μ*_*B*_^*γ*^)/*RT*] = 1−*a*_*B*_^*γ*^/*a*_*II*_^*β*^. Let us also denote *D*/[*V*_*m*_∙*r*_*I*_∙exp(−*Q*_*I*_/*RT*)] as *φ*_*I*_, and *D*/[*V*_*m*_∙*r*_*II*_∙exp(−*Q*_*II*_/*RT*)] as *φ*_*II*_: clearly *φ*_*I*_ and *φ*_*II*_ represent the ratios of diffusion rate over interface reaction rates, and have a unit of meter. Then Eqs. (2) and (3) can be respectively written as:


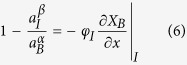



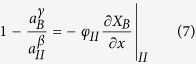


### Analytic and Numerical Solutions

Let us first consider two extreme cases. First, for the case that the interface reaction rates are much higher than the diffusion rate across the β layer (i.e., diffusion-rate limited), *φ*_*I*_ and *φ*_*II*_ are near zero; according to Eqs. (2)–(3), 3, *μ*_*B*_s are continuous at both interfaces, so *X*_*II*_^*β*^ = *X*_*II*_^*β,eq*^. Suppose *μ*_*B*_^*α*^ and the position of interface I, *x*_*I*_, are both constant with time, then *X*_*I*_^*β*^ is also constant, and the solutions to Eqs. (1) and (5) are respectively *X*_*B*_(*x*, *t*) = *X*_*I*_^*β*^−(*X*_*I*_^*β*^−*X*_*II*_^*β,eq*^)∙erf{(*x*−*x*_*I*_)/[2√(*Dt*)]}/erf(*k*/2) and *l *=* k*√(*Dt*) for the β layer, where *k* can be numerically solved from *k*∙exp(*k*^2^/4)∙erf(*k*/2) = 2/√π∙(*X*_*I*_^*β*^−*X*_*II*_^*β,eq*^)/*X*_*II*_^*β,eq*^. For instance, for *X*_*I*_^*β*^ = 0.26 and *X*_*II*_^*β,eq*^ = 0.17, *k *=* *0.953. On the other hand, if the interface reaction rates are much lower than the diffusion rate across β (e.g., as the β layer is thin), *φ*_*I*_ and *φ*_*II*_ are large; according to Eqs. (2) and (3), *X*_B_ and *J*_*B*_ are nearly constant in the entire β layer. Thus, (1−*a*_*B*_/*a*_*B*_^*α*^)/*φ*_*I*_ = (1−*a*_*B*_^*γ*^/*a*_*B*_)/*φ*_*II*_, from which *a*_*B*_ can be calculated. Integration of Eq. (5) gives: *l* ∝ *t*, and the pre-factor depends on the interface reaction rates.

For a general case between these two extremes, the equations can only be solved with the *μ*(*X*) relations of α and β provided. Next, let us consider a compound with a narrow composition range, so that as a Taylor series expansion is performed around *X*_*II*_^*β,eq*^ for its *a*(*X*_*B*_) curve, high-rank terms can be neglected because |*X*−*X*_*II*_^*β,eq*^| ≤ (*X*_*I*_^*β,eq*^−*X*_*II*_^*β,eq*^) is small; that is, *a*_*X*_ ≈ *a*_*B*_^*γ*^ + *κ*(*X*−*X*_*II*_^*β,eq*^), where *κ* is the linear coefficient of the *a*(*X*) curve. Given the complex boundary conditions for Eq. (1), to obtain the analytic solutions we introduce a second approximation if the β composition range is narrow: the *X*(*x*) profile of the β layer is linear so that at a certain time *J* is constant with *x*, such that −(∂*X*_B_/∂*x*)*|*_I_ ≈ −(∂*X*_B_/∂*x*)*|*_II_ ≈(*X*_*I*_^*β*^−*X*_*II*_^*β*^)/*l*. With these two approximations, we can solve Eqs. (6) and (7) and obtain that:


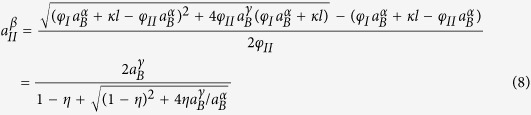


where *η *=* φ*_*II*_*a*_*B*_^*α*^/(*φ*_*I*_*a*_*B*_^*α*^+*κl*). Then *a*_*I*_^*β*^ can be calculated from *a*_*II*_^*β*^, and *X*_*I*_^*β*^ and *X*_*II*_^*β*^ can be calculated from *a*_*I*_^*β*^ and *a*_*II*_^*β*^ using *X* = *X*_*II*_^*β,eq*^ +(*a*_*X*_ − *a*_*B*_^*γ*^)/*κ*.

**Figure 3:**
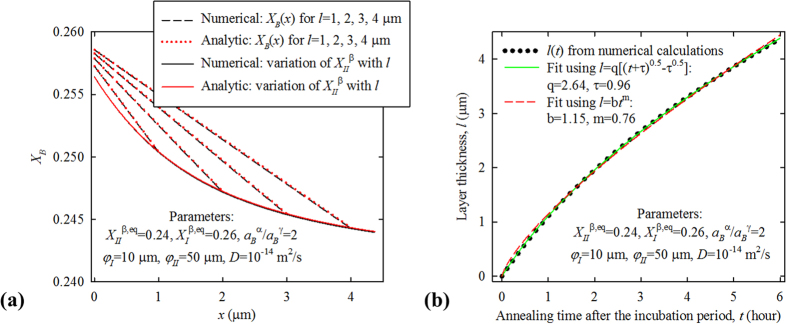
(**a**) The calculated *X*_*B*_(*x*) profiles of the hypothetical system for the analytic and numerical solutions, with and without the assumption that *X*_*B*_(*x*) is linear, respectively. (**b**) The *l*(*t*) results from the numerical calculations, with the fits of *l *=* q*[√(*t*+*τ*)−√*τ*] and *l *=* bt*^*m*^.

To verify the results, the equations are solved for a hypothetical system analytically and numerically, with and without the assumption that *X*(*x*) is linear, respectively. The obtained composition profiles are shown in Fig. 3(a). For simplicity, *μ*_*B*_^*α*^ of the system is set as *μ*_*B*_(A−*X*_*I*_^*β,eq*^ B) and is constant (for Nb_3_Sn systems, this means that Nb_6_Sn_5_ serves as Sn source), and the other parameters are specified in the figure. The difference between the analytic and numerical solutions is <0.1%, showing that the approximation of linear *X*(*x*) is good if the composition range is small (2 at.% in this case). The *l*(*t*) result (where *t* is the annealing time after the incubation period) from the numerical calculations is shown in Fig. 3(b). While the analytic *l*(*t*) solution is complicated, some *l*(*t*) relations with simple forms can be used as approximations. The most widely used *l*(*t*) relation for the case of constant *μ*_*B*_^*α*^ is *l *=* bt*^*m*^, in which *m *=* *1 for reaction-rate limited and *m *=* *0.5 for diffusion-rate limited; however, a defect with this relation is that as *l* increases from zero, it may shift from reaction-rate limited to diffusion-rate limited, so *m* may vary with *t*. Here a new relation *l *=* q*[√(*t*+*τ*)−√*τ*] (where *q* is a growth constant and *τ* is a characteristic time) is proposed. Such a relation is consistent with *l*^2^/*v*_*1*_+*l*/*v*_*2*_* *=* t* (where *v*_*1*_ and *v*_*2*_ are constants related to diffusion rate and interface reaction rates, respectively) proposed by previous studies^14^,^15^. This relation overcomes the above problem because as *t* ≪*τ*, *l  *=* *[*q*/(2*τ*)]∙*t* and as *t*≫ *τ*, *l *=* q*√*t*. As can be seen from Fig. 3(b), a better fit to the numerical *l*(*t*) curve in the whole range is achieved by *l *=* q*(√(*t*+*τ*)−√*τ*).

## Results and Discussion

Before discussing the application of this model to a specific material system, it must be pointed out that all of the analysis and calculations above are for the case that B diffuses through β bulk. In such a case, for an α/β/γ system, as *μ*_*B*_^*α*^ drops with the growth of β layer, *X*_*B*_(*x*) of the entire β layer should decrease with *μ*_*B*_^*α*^, because *μ*_*B*_^*α*^ ≥ *μ*_*I*_^*β*^ ≥ *μ*_*II*_^*β*^ ≥ *μ*_*B*_^*γ*^. Finally, one of two cases will occur: either *μ*_*B*_^*α*^ drops to *μ*_*B*_^*γ*^ (if A is in excess) so the system ends up with the equilibrium among γ, A–*X*_*II*_^*β,eq*^ B, and M–*X*_*1*_ B (as shown by the shaded region in the isothermal M–A–B phase diagram in Fig. 2), or γ is consumed up and β gets homogenized with time and finally *μ*_*B*_(β)* *=* μ*_*B*_(α) (as shown by the dashed line in Fig. 2). In either case, β layer eventually reaches homogeneity.

**Figure 4:**
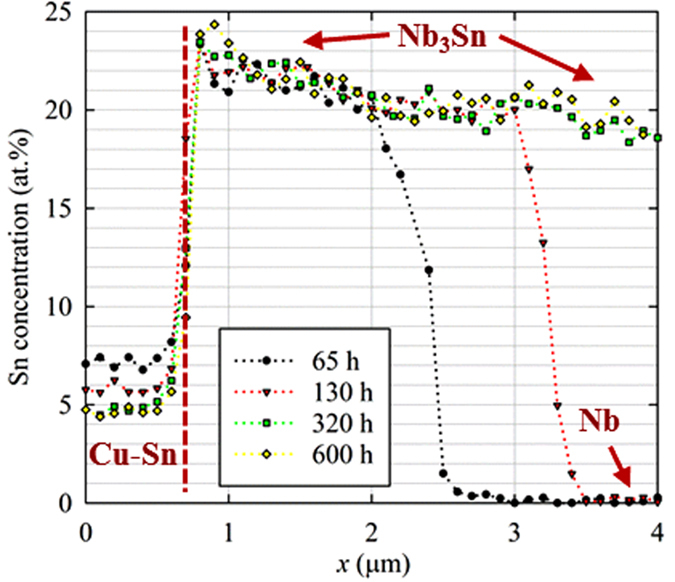
The measured *X*_*Sn*_s of a Cu-Sn/Nb_3_Sn/Nb system after various annealing times at 650 °C. The standard deviation in the Sn content measurements is around ±0.5 at.%.

However, we find that the composition could be different for a compound in which the bulk diffusion is low while grain boundary diffusion dominates. One such example is Nb_3_Sn, the composition of which displays some extraordinary features. As an illustration, the *X*_*Sn*_s of a Cu-Sn/Nb_3_Sn/Nb diffusion reaction couple after various annealing times are shown in Fig. 4. Clearly, as the *X*_*Sn*_ (and *μ*_*Sn*_) of Cu-Sn drop with time, the *X*_*Sn*_s of Nb_3_Sn do not drop accordingly; instead, they more or less remain constant with time. In addition, from 320 hours to 600 hours, although Nb has been fully consumed, the *X*_*Sn*_ of Nb_3_Sn does not homogenize (i.e., the *X*_*Sn*_ gradient does not decrease) with time. In many other studies on Cu-Sn/Nb systems with Nb in excess (e.g.,^4^,^9^,^10^), even after extended annealing times after the Nb_3_Sn layers have finished growing (which indicates that the Sn sources have been depleted, i.e., *μ*_*Sn*_s have dropped to *μ*_*B*_^*γ*^), *X*_*Sn*_s of Nb_3_Sn remain high above *X*_*II*_^*β,eq*^, without dropping with annealing time.

**Figure 5:**
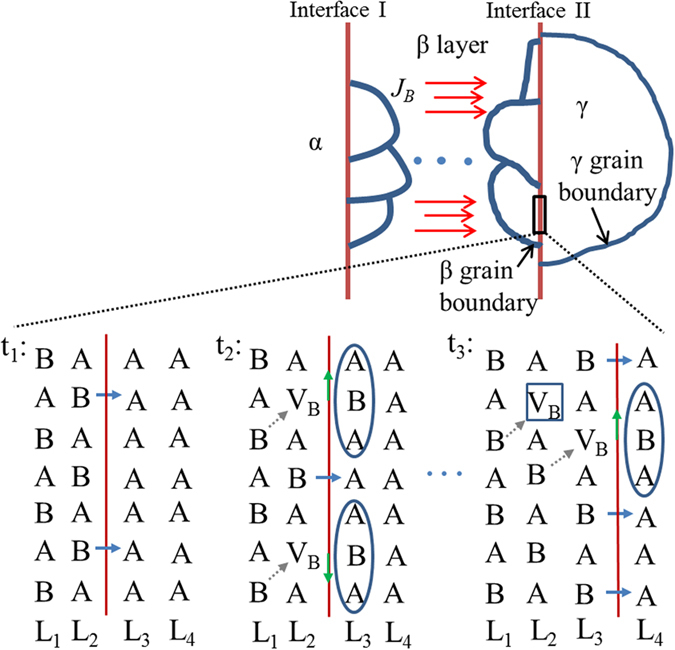
A schematic of the diffusion reaction process for grain boundary diffusion.

The reason for these peculiarities is that grain boundary diffusion in Nb_3_Sn dominates due to extremely low bulk diffusivity (e.g., lower than 10^−23^ m^2^/s at 650 °C)^21^,^26^,^27^ and small Nb_3_Sn grain size (~100 nm). In this case, our model and equilibrium-state analysis apply only to the diffusion zones (i.e., the grain boundaries and the inter-phase interfaces) instead of the bulk. To clarify this point more clearly, a schematic of the diffusion reaction process is shown in Fig. 5. At time *t*_*1*_, at the β/γ interface, high-B A_n_B (L_2_ layer) reacts with γ (L_3_ layer) to form some new A_n_B cells, leaving B vacancies (noted as V_B_s) in L_2_ layer (time *t*_*2*_). If bulk diffusivity is high, V_B_s simply diffuse through bulk (e.g., from L_2_ to L_1_, as shown by grey dotted arrows) to the B source. If bulk diffusion is frozen, the V_B_s diffuse first along β/γ inter-phase interface (as shown by green solid arrows), and then along β grain boundaries to the B source. This process continues until this L_3_ layer entirely becomes A_n_B (time *t*_*3*_), so the reaction frontier moves ahead to L_3_/L_4_, while the L_2_/L_3_ interface now becomes an inter-plane inside A_n_B lattice. If bulk diffusion is frozen, the V_B_s in the L_2_ layer that have not diffused to B source will be frozen in this layer forever, and will perhaps transform to A-on-B anti-site defects later (e.g., for Nb_3_Sn, Nb-on-Sn anti-sites are more stable than Sn vacancies^28^). Since these point defects determine the A_n_B composition, the *X*_*B*_ in this L_2_ layer cannot change anymore regardless of *μ*_*B*_ variations in grain boundaries. That is to say, *X*_*B*_ of any point is just the *X*_*II*_^*β*^ of the moment when the reaction frontier sweeps across this point, i.e., the *X*_*B*_(*x*) of the β layer is simply an accumulation of *X*_*II*_^*β*^s with *l* increase. Returning to Fig. 3(a), the dashed lines display the evolution of *X*_*B*_(*x*) with *l* increase for bulk diffusion, while that for grain boundary diffusion is shown by the solid lines. Since the energy dispersive spectroscopy (EDS) attached to scanning electron microscopes (SEM) that is used to measure the compositions typically has a spatial resolution of 0.5–2 μm, and thus mainly reflects the bulk composition, the composition characteristics of Nb_3_Sn layers as described above can be explained. It should be noted that knowledge of the difference between bulk diffusion and grain boundary diffusion is important in controlling the final composition of a compound. For instance, if bulk diffusivity is high, one method to form high-B A_n_B is increasing the starting B/A ratio so that after long annealing time for homogenization subsequent to the full consumption of A, *μ*_*B*_(α)* *=* μ*_*B*_(A−*X*_*I*_^*β,eq*^ B). However, our experiments demonstrate that for compounds with low bulk diffusivity (e.g., Nb_3_Sn), such an approach does not work; instead, controlling the *X*_*II*_^*β*^s while the compounds are growing is the only way. For those compounds with low but non-negligible bulk diffusivities, their compositions would be between these two extremes.

Then what determines the bulk composition as grain boundary diffusion dominates? From Fig. 5, it can be clearly seen that there is a competition deciding the V_B_ fraction in the frontier A_n_B layer: at *t*_*2*_ the reaction across the β/γ interface produces V_B_s in L_2_ layer, while the diffusion of B along β grain boundaries and α/β interface fills these V_B_s. Thus, if the diffusion rate is slow relative to the reaction rate at interface II (i.e., *φ*_*II*_ is low), a high fraction of V_B_s would be left behind as the interface II moves ahead, causing low B content; if, on the other hand, the diffusion rate is high relative to the reaction rate at interface II, the A_n_B layer has enough time to get homogenized with the B source, causing low *X*_*B*_ gradient. In this case, the *μ*_*B*_ of B source and the reaction rate at interface I together set a upper limit for *μ*_*B*_ of β.

**Figure 6:**
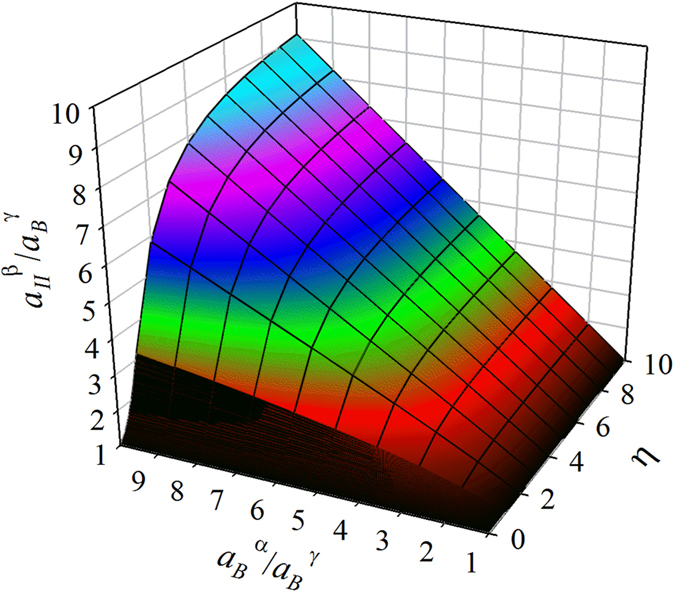
The variation of *a*_*II*_^*β*^ with *η* and *a*_*B*_^*α*^, according to Eq. (8).

Next, we will modify the above model for the case of grain boundary diffusion for quantitative analysis. As pointed out earlier, the chemical potentials of grain boundaries can change with *μ*_*B*_^*α*^ and *l*, while those of the bulk cannot. In such a case, *μ*_*I*_^*β*^ and *μ*_*II*_^*β*^ (suppose the diffusivities along the inter-phase interfaces are large) can still be calculated using our model, provided that the *μ*(*X*) relation and *D* of the β grain boundary (instead of the bulk) are used in all of the equations, and that *φ*_*I*_ and *φ*_*II*_ are multiplied by a factor of ∑*A*_*GB*_/*A*_*int*_ (where ∑*A*_*GB*_ is the sum of the cross section areas of the grain boundaries projected to the inter-phase interfaces), because B diffuses only along β grain boundaries while reactions occur at the entire interfaces. Approximately, ∑*A*_*GB*_/*A*_*int*_ ≈ [1−*d*^2^/(*d* + *w*)^2^] ≈ 2*w*/*d* (where *w* is the β grain boundary width, and *d* is the grain size). Apparently, grain growth with annealing time reduces the diffusion rate. According to Eq. (8), *a*_*II*_^*β*^ is determined by *η* and *a*_*B*_^*α*^, and increases with them, as shown by Fig. 6. Since *η *=* φ*_*II*_*a*_*B*_^*α*^/(*φ*_*I*_*a*_*B*_^*α*^ + *κl*)* *=* *1/[*φ*_*I*_/*φ*_*II*_ + *κl*/(*φ*_*II*_*a*_*B*_^*α*^)], clearly *η* decreases as *φ*_*I*_/*φ*_*II*_ and *l* increase, and the influence of *l* (which reflects the *X*_*II*_^*β*^-*x* gradient) is mitigated as *φ*_*II*_*a*_*B*_^*α*^ increases. Thus, to improve *X*_*II*_^*β*^ of A_n_B at *l *=* *0, one should increase *μ*_*B*_^*α*^ and the reaction rate at interface I, and reduce the reaction rate at interface II; meanwhile, to reduce *X*_*II*_^*β*^(*x*) gradient, one should increase *φ*_*II*_ (which means improving the diffusion rate or reducing the reaction rate at interface II) and *a*_*B*_^*α*^. Apparently, these quantitative conclusions are consistent with the above qualitative analysis.

**Figure 7:**
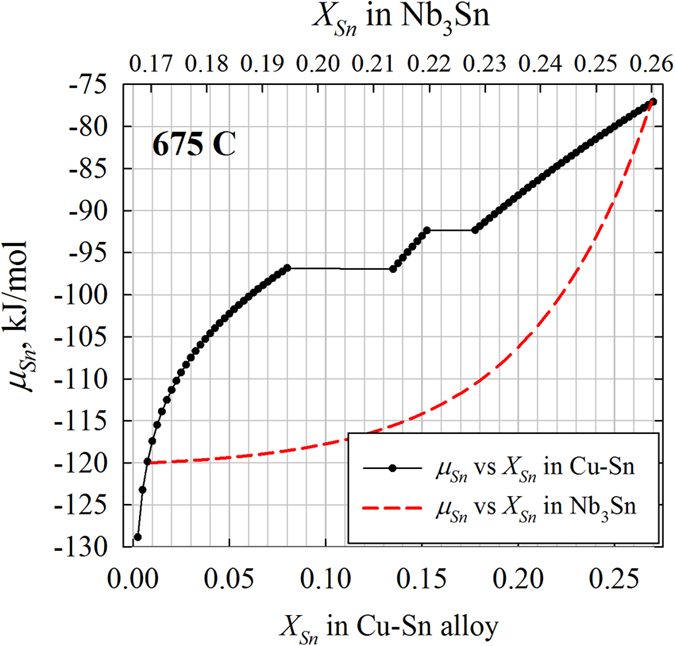
The variation of *μ*_*Sn*_ with *X*_*Sn*_ for Cu-Sn calculated based on thermodynamic data given in ref. 30, and a rough, speculative *μ*_*Sn*_(*X*_*Sn*_) relation for Nb_3_Sn sketched according to the phase formation relation between Cu-Sn and Nb_3_Sn.

Next let us compare this model with the example of Nb_3_Sn. It has been well established from experimental work that there are mainly two factors that can significantly influence the Sn content of Nb_3_Sn in a Cu-Sn/Nb_3_Sn/Nb diffusion reaction couple: heat treatment temperature and Cu-Sn source. The heat treatment temperature can simultaneously influence multiple factors of Eq. (8), such as *a*_*B*_^*α*^, *D*, and reaction rates at both interfaces, etc. Thus, the explanation of the influence of temperature on Sn contents using this theory requires knowledge of the quantitative variations of these factors with temperature. For the other factor, Cu-Sn source, the diffusion reaction couples can be classified into two types based on the Cu-Sn source: the type I uses bronze (with Sn content in Cu-Sn typically below 9 at.%) as Sn source, and the type II uses high-Sn Cu-Sn (e.g., Cu-25 at.% Sn). Previous measurements^4^,^9^,^10^,^11^,^29^ show that both types of samples have Sn contents above 24 at.% for the Nb_3_Sn layer next to the Cu-Sn source; however, they have distinct Sn content gradients as the Nb_3_Sn layers grow thicker: the type I generally has Sn content gradients above 3 at.%/μm^29^, while those of the type II are below 0.5 at.%/μm^4^,^9^,^10^. Such a difference in the Sn content gradients leads to distinct grain morphologies and superconducting properties. The different *X*_*Sn*_ gradients in the two types of samples with different Cu-Sn sources can be easily explained by our theory above: according to Eq. (8), increased *μ*_*B*_^*α*^ can decrease *X*_*Sn*_ gradients. It may also need further investigation regarding whether Cu-Sn source can also influence diffusion rates in Nb_3_Sn layer (e.g., via thermodynamic factor), because greater *D* leads to greater *φ*_*II*_, which helps decreasing *X*_*Sn*_ gradients. As to the phenomenon that different wires have similar *X*_*Sn*_ in the Nb_3_Sn layer next to the Cu-Sn source, the relation between *μ*_*Sn*_(Cu-Sn) and *μ*_*Sn*_(Nb-*X*_*Sn*_ Sn) is required. The Cu-Sn system has been well studied, and the phase diagram calculated by the CALPHAD technique using the thermodynamic parameters given by ref. 30 is well consistent with the experimentally measured diagram^31^. Thus, the parameters from ref. 30 are used to calculate *μ*_*Sn*_ of Cu-Sn, which is shown in Fig. 7. On the other hand, although thermodynamic data of Nb-Sn system were proposed by refs. 30 and 32, in these studies Nb_3_Sn was treated as a line compound. However, some information about *μ*_*Sn*_ of Nb_3_Sn can be inferred from its relation with *μ*_*Sn*_ of Cu-Sn: since Cu-7 at.% Sn leads to the formation of Nb-24 at.% Sn near the Cu-Sn source^29^, we have *μ*_*Sn*_(Cu-7 at.% Sn) ≥ *μ*_*Sn*_(Nb-24 at.% Sn). Thus, the expected approximate *μ*_*Sn*_(Nb-*X*_*Sn*_ Sn) curve in a power function is shown in Fig. 7. Furthermore, we can also infer that the Sn transfer rate at the Cu-Sn/Nb_3_Sn interface must be much faster than that at the Nb_3_Sn/Nb interface, so *μ*_*Sn*_ discontinuity across the interface I is small. These explain why low-Sn Cu-Sn can lead to the formation of high-Sn Nb_3_Sn. It is worth mentioning that from Fig. 7, it is clear that the Taylor series for the true *a*(*X*) relation of Nb_3_Sn have more high-rank terms than *a*(*X*)≈*a*_*B*_^*γ*^ + *κ*(*X*−*X*_*II*_^*β,eq*^); however, our numerical calculations show that adding high-rank terms to the *a*(*X*) relation does not lead to different conclusions regarding the influences of *a*_*B*_^*α*^, *φ*_*I*_, *φ*_*II*_, and *l* on *X*_*II*_^*β*^. Thus, the above qualitative and quantitative analysis still applies.

In summary, a mathematical framework is developed to describe the compositions and layer growth rates of non-stoichiometric intermediate phases formed by diffusion reactions. The governing equations are derived and analytic solutions are given for compounds with narrow composition ranges under certain approximations. We also modify our model for compounds in which bulk diffusion is frozen, of which the bulk is not in equilibrium with the rest of the system. Based on this model, the factors that fundamentally determine the compositions of non-stoichiometric compounds formed by diffusion reactions are found and approaches to control the compositions are proposed.

## Methods

For the Cu-Sn/Nb_3_Sn/Nb diffusion reaction couples that were used for Sn content measurements (the results of which are shown in Fig. 4), the initial composition of the precursor Cu-Sn alloy was Cu-12 at.% Sn. The samples were reacted at 650 °C for 65 h, 130 h, 320 h, and 600 h. Then the surfaces of the samples were polished to 0.05 μm and the compositions were measured using an EDS system attached to an SEM. An accelerating voltage of 15 kV was used for the quantitative line scans. A standard Nb-25 at.% Sn bulk sample provided by Dr. Goldacker from Karlsruhe Institute of Technology was used for calibrating the Sn content of the samples. The standard deviation in the measurements was found to be about ± 0.5 at.%.

## Additional Information

**How to cite this article**: Xu, X. and Sumption, M. D. A model for the compositions of non-stoichiometric intermediate phases formed by diffusion reactions, and its application to Nb_3_Sn superconductors. *Sci. Rep.* **6**, 19096; doi: 10.1038/srep19096 (2016).
